# A Non-Isothermal Pore Network Model of Primary Freeze Drying

**DOI:** 10.3390/pharmaceutics15082131

**Published:** 2023-08-14

**Authors:** Maximilian Thomik, Felix Faber, Sebastian Gruber, Petra Foerst, Evangelos Tsotsas, Nicole Vorhauer-Huget

**Affiliations:** 1 Thermal Process Engineering, Institute of Process Engineering, Otto-von-Guericke University Magdeburg, Universitaetsplatz 2, 39106 Magdeburg, Germany; felix.faber@ovgu.de (F.F.); nicole.vorhauer-huget@ovgu.de (N.V.-H.); 2 Food Process Engineering, TUM School of Life Sciences, Technical University of Munich, Weihenstephaner Berg 1, 85354 Freising, Germany

**Keywords:** primary freeze drying, pore-scale resolved simulation, coupled heat and mass transfer, 3D domain, regular square lattice, sublimation front position, time- and space-dependent temperature, pressure and flow rates

## Abstract

In this work, a non-isothermal pore network (PN) model with quasi-steady vapor transport and transient heat transfer is presented for the first time for the application of primary freeze drying. The pore-scale resolved model is physically based and allows for the investigation of correlations between spatially distributed structure and transport conditions. The studied examples were regular PN lattices with a significantly different structure, namely a spatially homogeneous PN, also denoted as monomodal PN, and a PN with significant structure variation, referred to as bimodal PN because of its bimodal pore size distribution. The material properties selected for the solid skeleton in this study are equivalent to those of maltodextrin. The temperature ranges applied here were −28 °C to −18 °C in the PN and −42 °C in the surrounding environment. The environmental vapor pressure was 10 Pa. The PNs were dried with constant temperature boundary conditions, and heat was transferred at the top side by the vapor leaving the PN. It is shown how the structural peculiarities affect the local heat and mass transfer conditions and result in a significant widening of the sublimation front in the case of the bimodal PN. The possibility of spatially and temporally resolved front structures is a unique feature of the PN model and allows the study of situations that are not yet described by classical continuum approaches, namely heterogeneous frozen porous materials. As demonstrated by the thin layers studied here, the pore-scale simulations are of particular interest for such situations, such as in lyomicroscopes or collagen scaffolds, where a length-scale separation between dry and ice-saturated regions is not possible.

## 1. Introduction

Freeze drying or lyophilization is an essential process for the preservation of pharmaceutical and biological products. It is carried out at temperatures and pressures well below the triple point of water.

Typical mathematical models of freeze drying have been developed for different scales: single particles, aggregates and biological products such as fruits and frozen suspensions in vials or syringes on the small scale, as well as particle beds or complete drying chambers on the large scale. While, on the small scale, the impact of freeze drying conditions on the drying time, temperature of the material and pressure are most often of interest, the large-scale approaches focus on the impact of the spatially and temporally varying process conditions. The latter usually require complex three-dimensional (3D) models that are often implemented in COMSOL [1], while in the first case, in most situations, 1D models are sufficient due to the anticipation of product homogeneity [2]. In such models, effective parameters are used and no spatial variation in the pore structure is considered.

In the literature, a wide range of model assumptions can be found, which differ not only in terms of postulated steady-state or transient conditions, but also in terms of the incorporated physical phenomena. The accuracy of the proposed models surely increases with higher complexity, provided that the used parameters are reliably determined, e.g., by experiments. However, the computational efficiency usually suffers from the number of physical mechanisms that are included or increasing size and dimension of the computational domain. Secondly, the accurate prediction of model parameters is challenging in most cases. This can be disadvantageous, especially in cases of model predictive control, e.g., [3]. The choice of the model is therefore most often a compromise between accuracy and computation time.

Here, we want to provide a brief literature overview in which we focus on the models that have been proposed for the primary freeze drying of frozen bulk solutions, such as suspensions that are frozen as blocks inside vials and syringes. The focus is thereby on the studies of single vials (sample scale), without excluding the work of, e.g., [4], where the outcomes from simulations of single vials are extrapolated to the drying chamber scale. Secondary drying (occurring before primary drying is finished, i.e., taking place in parallel) is often neglected in these models.

Primary freeze drying is generally constrained by the process conditions, namely pressure and temperature, which dictate the limits of product integrity and therefore restrict drying time and energy efficiency [5,6]. Temperature, pressure and drying time are thus the essential parameters that are usually predicted, e.g., for process control, e.g., [3]. The most critical parameter is thereby clearly the product temperature, which needs to be low enough to avoid material denaturation, melting or product collapse throughout the vial and during the whole drying process [6]. It depends on the temperature of the shelf and the pressure of the chamber, which both also control the drying rate.

In the 2D dusty gas model presented in [7], transient heat transfer and coupled mass transfer are solved. In this, the dried region is considered as a porous medium with constant porosity; thermal conductivity, density and heat capacity are averaged for the mixture of solid and empty pores. In the frozen region, these parameters are averaged for water ice and solids. Here, only the top surface is assumed permeable for vapor transfer, whereas all other sides are impermeable. Mass transfer is in most cases computed either as a diffusion problem or as viscous flow, the latter often in presence of an inert gas. For example, [7,8] proposed to use the Darcy equation with the permeability of the porous dried region for vapor transfer simulation.

The heat transfer equations, including the transient situation of the warming-up or cooling-down of the material (i.e., the sensible heat), are set up and solved for both dried and frozen regions. In most cases, a moving boundary condition (Stefan problem [4,7]) is postulated based on the temporal variation in the sublimation interface, where thermodynamic equilibrium between ice and vapor phase is assumed. The equilibrium vapor pressure is mostly calculated with the Clausius–Clapeyron equation or with the equation from Goff and Gratch (1951) [9]. The heat supply is usually realized by conduction through the vial bottom, while at the top, surface heat transfer by radiation from the environment can be assumed, e.g., [10]. Several proposals have been made for the heat exchange with the environment along the side walls, which include radiation and conduction, e.g., [3]. Heat transfer inside the frozen region is based on conduction through the ice–solid mixture; in the dried region, heat transfer through the interconnected solid walls as well as heat convection are assumed [11]. At very low chamber pressure (below 10 Pa and in the absence of inert gases), the problem is simplified and heat convection is disregarded, e.g., [1].

The assumption of homogeneous domains that are assigned with effective, i.e., averaged properties, usually form the basis for further simplification of the problem to 1D, i.e., along the vertical height of the blocks [11]. This is surely accurate enough, when it can be expected that the sublimation of ice results in the formation of a flat (infinitesimally thin) phase boundary at the top side of the frozen block which moves downwards during the drying process. In other cases, i.e., when either structural heterogeneity or heat transfer in the lateral direction cannot be disregarded, the sublimation front can be convex (curved downwards along the side walls of the vial) [2], or concave [12].

Even if the dimension of the domain is reduced to the space coordinate in the vertical direction, the computational effort is still high, making this approach uncomfortable for online process control [3]. As a consequence, less-complicated model approaches with simplified assumptions for both heat and mass transfer were developed [4]. Often in these models, a fitting function for the mass transfer, depending on a priori conducted experiments, is used [3,4,13]. Assuming quasi-stationary heat transfer, the partial differential equation (from the transient case with a first order derivative over time and second order derivative over space) is transferred into an ordinary differential equation. This reduces the computational effort significantly without altering the simulation results too much. Alternatively, the analytical solution of the transient heat transfer problem is used [4]. It was shown in the same study that the simulation results fit well with the experimental results. The overall agreement with other studies, e.g., [14], is good. The simplified model proposed in [3] is furthermore used with the model-based determination of mass and heat transfer coefficients with the purpose of determining the limits of primary freeze drying in terms of maximum temperature and chamber pressure [6]. The in silico determination of model parameters is an alternative option to experimental measurements, which are often elaborate, time consuming and less accurate [6]. As an important feature, these coefficients can be randomly distributed for the simulations, thus allowing us to predict a wide range of drying behavior in single vials under different conditions, as it can be expected for the vials on the shelf in a drying chamber [13].

This brief literature overview shows that the mathematical models that describe primary freeze drying of frozen bulk solutions consider the vial contents as a continuum with averaged effective parameters, such as effective porosity, thermal conductivity, heat capacity and transport coefficients. As an example, heat transfer coefficients can incorporate several different effects, including heat transfer by conduction through the solid and ice phase, heat transferred by vapor or heat exchange with the vial and the surroundings. The available approaches are intended to predict the global variation of, e.g., drying time in dependence of the process conditions, i.e., temperature and pressure, which can vary along the position of the vials on the shelf. Thus, they provide data about the inter-vial heterogeneity of freeze drying, which is important for process and apparatus design and process control. However, these models are not suitable for the investigation of intra-vial heterogeneity which is linked to the local, and sometimes even temporal, variation in material properties. The authors of [15] studied the permeability of the pore structure in dependence of cooling rate based on Darcy’s flow equation. They found that smaller pores and thus smaller permeabilities of the vapor phase are obtained at higher cooling rates, where the nucleation temperature is smaller. A similar finding was recently reported in [16] where higher nucleation temperatures were associated with larger ice crystals. This shows the need for the development of models for heterogeneous and composite materials [17].

To the best of our knowledge, this is the first time that a non-isothermal pore network model (PNM) is presented for the mathematical modeling of primary freeze drying. In contrast to the averaging approaches, the PNM resolves the structure of the material on the pore scale. It can therefore consider local effects, such as the variation in pore sizes, which have a direct impact on the local heat and mass transfer conditions. PNMs thus serve for the study of structure–transport correlations, which is already widely established for conventional drying methods [18]. There, the capillarity of porous media yields strongly structured drying fronts which again affect the drying kinetics. Although capillary transport does not play a role in freeze drying, the heat and mass transfer conditions can alter the sublimation front structure. The use of PNMs can thus be useful to understand and predict the drying kinetics of heterogeneous materials, such as graded or foamed structures, or to study freeze drying at the limits of material collapse. In cases where a length-scale separation is not possible, e.g., in thin porous layers, the use of PNMs may even be mandatory [19].

With the proposed simulation tool, freeze drying can be studied on a physical base. Temperature, pressure and ice content are locally resolved. Besides this, it yields effective parameters as a function of ice content, which could be used for the improved parameterization of the above-described approaches [13]. In particular, the local heat and mass transfer coefficients of heterogeneous porous media could be adjusted based on PNM studies. Only a few parameters are involved that need to be a priori defined, and these parameters concern the boundary conditions of heat and mass transfer as well as the material’s morphology. The PNM can be realized with regular lattices, e.g., as proposed in [20], or reconstructed porous domains can be implemented, e.g., [21]. The size of the domain and the incorporated physical mechanisms thereby dictate the computational effort.

One major advantage of PNM is its ability to study the impact of structure on heat and mass transfer conditions independently of each other. This makes fundamental physical mechanisms much more accessible.

The approach described here can be used to study structure–transport relationships that can be helpful to optimize freeze drying in terms of drying time and energy consumption [5,13]. More clearly, it can be studied if heat and/or mass transfer can be improved by a change in geometry, e.g., height or width, as well as by an increase in pore sizes and/or porosity, which might be adjusted according to the freezing conditions or the composition of the mixture.

## 2. Materials and Methods

The non-isothermal PNM of primary freeze drying is a further development of the model in [22]. In contrast to the previous model, the computation of mass transfer is only considered for the vapor phase and in the transition regime between molecular (Knudsen) diffusion and viscous flow. The water is contained inside the void space in the frozen state, for which capillary liquid pumping is not computed. Transient heat transfer based on conduction through the solid and ice phases is solved using the Krischer Model [23]. In addition to that, heat transfer by vapor flow through empty pores, i.e., advection, is implemented in the new model.

### 2.1. Pore Network Model

**Figure 1:**
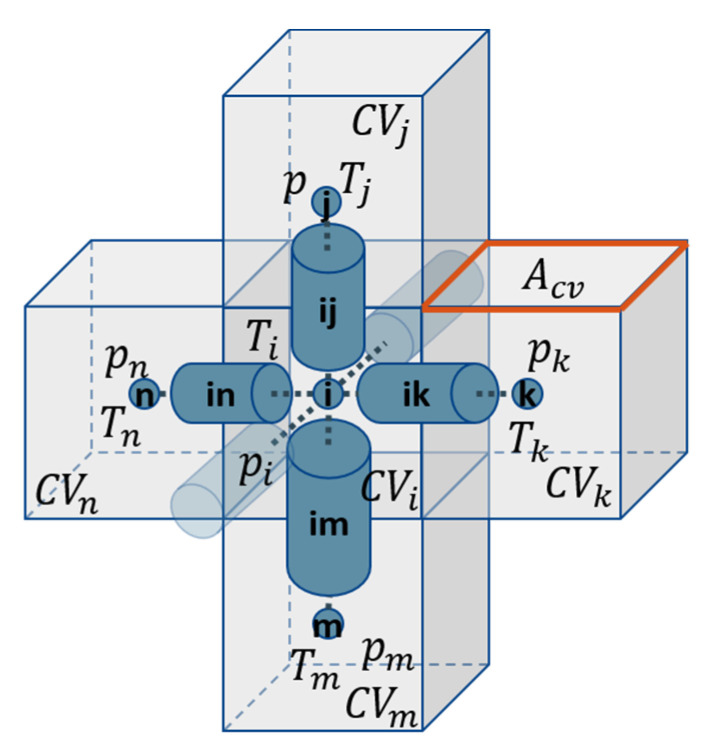
Schematic view of the regular square lattice. The computational nodes, assigned by letters *i*-*n*, contain no volume.

The simulations are conducted using regular 3D square lattices. The network is realized as a bond network, which means that the void space is completely assigned to cylindrical pores that are connected by computational nodes without volume (Figure 1). While the structural and physical properties, such as length, width, cross-section and mass transfer resistance are allocated to the pores, the computational nodes are used to solve the enthalpy and mass balances and to store the pressure data (mass balances) as well as the temperature data (enthalpy balances).

As shown in Figure 1, each computational node is surrounded by a control volume (CV). It is cubic in the case of the regular PN studied here. The edge length is constant and identical with the pore length, i.e., $L_{CV}=L_{ij}$, the volume is $V_{CV}=L_{CV}{}^{3}$ and the contact area between two neighboring CVs is $A_{CV}=L_{CV}{}^{2}$ in this case. The ice phase is equally distributed in partially saturated pores.

The CVs are assigned with thermo-physical properties (thermal conductivity and heat capacity) as well as volume, porosity and density. Thermal conductivity, heat capacity and density depend on local saturation as well as porosity, and are updated during the simulation.

**Figure 2:**
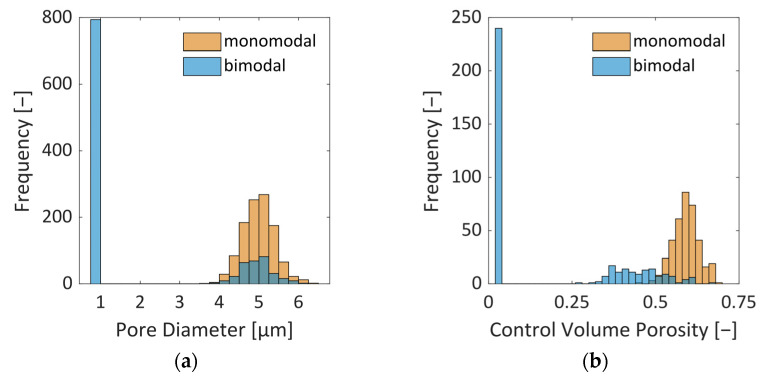
(**a**) Distribution of pore diameters in the two PNs studied here. The PN with bimodal PSD has a peak (without spread) at 1 µm and a peak at 5 µm; whereas the PN with monomodal PSD has only one peak at 5 µm. (**b**) Distribution of CV porosity as computed from Equation (1). The peaks are found at 0.6 for the monomodal PSD and range from 0.25 to 0.7, and an additional peak is located at 0.02 for the bimodal PSD.

An overview of the geometric parameters of the PNs considered in this work is provided in Table 1. The pore sizes are normally distributed (Figure 2) based on structural analysis carried out in [21,24]. In addition to a PN with monomodal pore size distribution (PSD), representing a homogeneous material, we consider the case of local variation in the PSD here. The second PN is therefore realized with a bimodal PSD. It has larger pores in the center and smaller pores along the sides. The ratio of small to large pores is 2.59. This means that the PN contains a much higher number of smaller pores. This mimics the situation of structural heterogeneity that could be expected from the freezing step [25]. Here, we assume that the lateral sides could be frozen faster with the result of smaller ice crystals than in the center, where we suppose larger ice crystals and pores exist.

Note that, in the bimodal PN, different CVs occur: (i) CVs in the very center which contain only pores with larger diameters, (ii) CVs at the very periphery which contain only pores with small diameters and (iii) CVs at the boundary of both regions which contain a mixture of larger and smaller pores.

**Figure 3:**
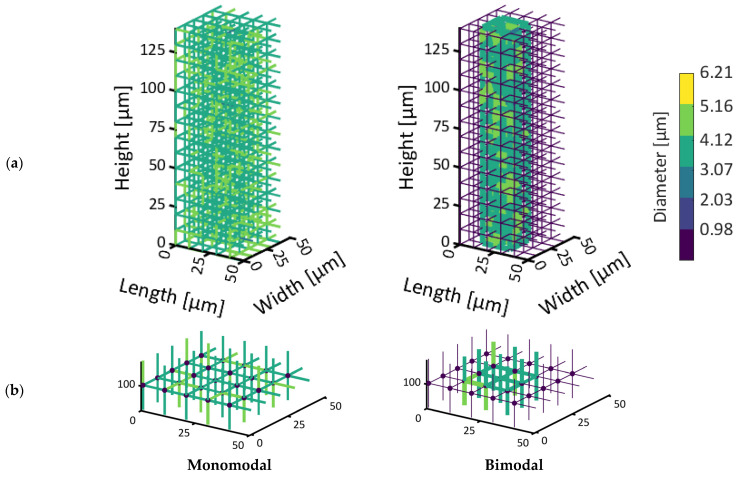
(**a**) Visualization of the two PNs under investigation. (**b**) Closer visualization of a slice at a height of 100 µm. The different colors of the pores are associated with their diameter as provided by the color scale. Dots represent computational nodes.

The PNs are shown in Figure 3. The image in Figure 3a represents the PN with monomodal PSD, while in Figure 3b, the PN with bimodal PSD is shown. The color code of the pores refers to their diameter. Brighter colors represent larger pores.

### 2.2. Model Assumptions and Parameter Settings

The PNM was developed for the simulation of primary freeze drying. The desorption of bound water, which is usually associated with secondary freeze drying, was not considered. The structural properties of the PN were kept constant, i.e., the void space and solid bridges were not altered, e.g., as a consequence of material shrinkage or collapse.

**Figure 4:**
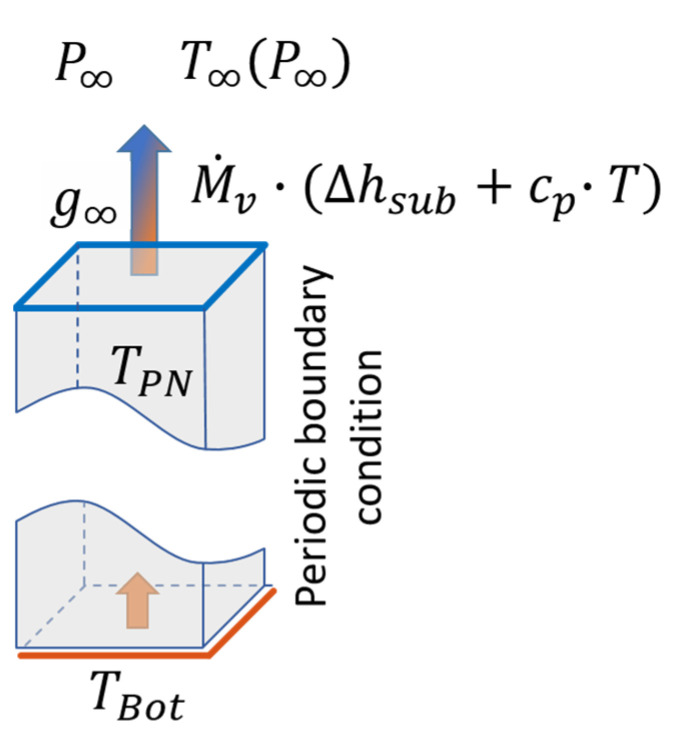
Schematic illustration of computational domain with boundary conditions.

The void space of the PN is initially completely saturated with frozen water. Sublimation is then initiated at the top side of the PN. The bottom side is made impermeable for mass transfer and periodic boundary conditions are applied at the sides. Heat is supplied by contact of the PNs to a virtual heating shelf at the bottom side, following the set-up in [26]. A constant temperature boundary condition is applied at this side. Radiative heat transfer is neglected in the simulations. The top side of the PN is virtually connected to the bulk of the freeze drying chamber, which is assigned with constant temperature (*T*_∞_) and pressure (*P*_∞_) (Figure 4). The process parameters are summarized in Table 2.

Table 3 summarizes the material properties. In this study, the solid is represented by maltodextrin with the material properties given in Table 3. As can be seen, the physical properties are assumed to be constant, i.e., independent of temperature and pressure.

### 2.3. Determination of the Properties of the Control Volumes

The geometry of the control volumes (CVs) is schematically illustrated in Figure 1. Depending on the local variation in the pore sizes, different values of porosity *ε*_CV_ (Figure 2b),

(1) $$\varepsilon_{CV}=\frac{\sum_{ij}^{io}V_{ij}/2}{L_{CV}{}^{3}}$$

effective density $\rho_{CV}$,

(2) $$\rho_{CV}=S_{CV}\cdot \varepsilon_{CV}\cdot \rho_{ice}+\left(1-\varepsilon_{CV}\right)\cdot \rho_{s},$$

effective heat capacity,

(3) $$c_{p}{}_{,CV}=S_{CV}\cdot \varepsilon_{CV}\cdot c_{p}{}_{,ice}+\left(1-\varepsilon_{CV}\right)\cdot c_{p}{}_{,s},$$

as well as effective thermal conductivity,

(4) $$\lambda_{CV}=\frac{1}{\frac{a}{\lambda_{ser,CV}}+\frac{1-a}{\lambda_{par,CV}}},$$

are computed. In Equation (4), the thermal conductivity in series is given by

(5) $$\lambda_{ser,CV}=\frac{1}{S_{CV}\frac{\varepsilon_{CV}}{\lambda_{ice}}+\frac{1-\varepsilon_{CV}}{\lambda_{s}}},$$

and the thermal conductivity in parallel is given by

(6) $$\lambda_{par,CV}=S_{CV}\cdot \varepsilon_{CV}\cdot \lambda_{ice}+\left(1-\varepsilon_{CV}\right)\cdot \lambda_{s}.$$

The final equation is different to previous works [22], where the orientation of pores was not considered. The factor a is assumed as *a* = 0.5 in this study. The saturation of the CVs is calculated from the sum of the ice volume divided by the overall void space of pores contained inside it:

(7) $$S_{CV}=\frac{V_{ice,CV}}{V_{void,CV}}.$$

Additionally, the saturation of single pores is calculated analogously.

The effective parameters thus depend both, on the porosity *ε_CV_* as well as on the saturation *S_CV_* of CVs with water ice.

### 2.4. Mass Transfer

The transfer of water vapor is computed based on the pressure gradients between computational nodes *i* and *j* that have at least one empty pore neighbor *ij* assuming quasi-steady conditions. The mass transfer resistance is thereby determined by the properties of the empty pores surrounding the nodes:

(8) $${\dot{M}}_{v,ij}=g_{ij}\cdot \left(P_{i}-P_{j}\right),$$

with $g_{ij}=\frac{A_{ij}\cdot {\tilde{M}}_{v}}{\tilde{R}\cdot T_{ij}\cdot L_{ij}}\cdot K_{ij}$ and temperature $T_{ij}=\frac{T_{i}+T_{j}}{2}$ computed as the arithmetic mean value of the temperatures stored in the adjacent computational nodes, *T_i_* and *T_j_*. The length *L_ij_* is the length of the pore, which can be either empty or partially saturated. Thus, the distance between the ice interface and the surface of the pore is not tracked in the current version of the model. Cylindrical cross sections,

(9) $$A_{ij}=\pi r_{ij}{}^{2},$$

are assumed.

In Equation (8), *K_ij_* is computed from the correlation [32],

(10) $$K_{ij}=\frac{d_{ij}^{2}\cdot P_{ij}}{32\cdot \eta_{v}}\left(1+8.88\cdot Kn_{ij}+4.96\cdot Kn_{ij}{}^{2}\right),$$

with $P_{ij}=\frac{P_{i}+P_{j}}{2}$ and Knudsen number

(11) $$Kn_{ij}=\frac{\Lambda_{ij}}{d_{ij}}.$$

In Equation (10), $K_{ij}\cdot \eta_{v}/P_{ij}$ denotes the permeability of the dry cake [32] and in Equation (11), the mean free path of the vapor in empty pores is computed under usage of the ideal gas constant $\tilde{R}=8.3145\;J/\mathrm{mol}/K$ from

(12) $$\Lambda_{ij}=\frac{\eta_{v}}{P_{ij}}\sqrt{\frac{\pi \cdot \tilde{R}\cdot T_{ij}}{2\cdot {\tilde{M}}_{v}}},$$

with the arithmetic mean values again used for temperature and pressure.

Applying the law of conservation of mass to each computational node,

(13) $$\sum_{i=1}^{n}{\dot{M}}_{v,ij}=0,$$

the pressure is available from the set of linear equations, with the number of equations identical to the number of computational nodes,

(14) $$A_{v}\cdot P_{i}=b_{v},$$

with the matrix of conductances,

(15) $$A_{v}=\left[\begin{array}{ccccc} & \vdots & & \vdots & \\ \cdots & \sum g_{ij} & \cdots & -g_{ik} & \cdots \\ & \vdots & & \vdots & \\ \cdots & -g_{ik} & \cdots & \sum g_{ki} & \cdots \\ & \vdots & & \vdots & \end{array}\right],$$

and the vector of boundary conditions,

(16) $$b_{v}=\sum g_{ij}\cdot P_{i}.$$

Equation (14) is solved numerically in MATLAB (R2022a) using the following boundary conditions. At the top side, a constant pressure *P*_∞_ is applied, which represents the pressure in the environment of the freeze drying PN. The connection between the surface pores with pressure *P*_i_ to virtual nodes with *P*_∞_ (Figure 4) is implemented with a constant mass transfer coefficient *g*_∞_:

(17) $${\dot{M}}_{v,i\infty }=g_{\infty }\cdot \left(P_{i}-P_{\infty }\right).$$

The value of *g*_∞_ = const. is assumed as *g*_∞_ = 1∙10^−12^ m∙s = const. for both PNs in this study. This value was chosen based on preliminary studies of both networks with the condition to not impede the mass transfer at the top side of the PN.

At the sublimation front, thermodynamic equilibrium is assumed and the pressure is calculated based on [9]:

(18) $$\log_{10}\left(P^{eq}\right)=-9.09718\left(\frac{T^{*}}{T}-1\right)-3.56654\cdot \log_{10}\left(\frac{T^{*}}{T}\right)+0.876793\left(1-\frac{T}{T^{*}}\right)+\log_{10}\left(P^{*}\right).$$

In this, T* = 273.16 K and P* = 6.1071 hPa are the temperature and pressure at the triple point of water, respectively. The temperature *T* is the regarding equilibrium temperature at the sublimation front. This links the mass transfer to the heat transfer problem.

### 2.5. Sublimation Rate

The local phase change or sublimation rate of each ice-filled pore along the gas–solid phase boundary is computed from the mass balances of the computational nodes that are located at the endings of sublimating pores. Because each pore lays between exactly two nodes, it is possible (especially for horizontal pores), but not necessarily a given, that sublimation occurs concurrently at both endings.

(19) $${\dot{M}}_{sub,ij}=\frac{A_{i}}{\sum_{i}A_{sub,i}}\sum_{i}^{}{\dot{M}}_{v,i}+\frac{A_{j}}{\sum_{j}A_{sub,j}}\sum_{j}^{}{\dot{M}}_{v,j}.$$

Here, only positive vapor flow rates are regarded to accumulate the sublimation rate. Any negative flow rates that might occur due to the flow of vapor from a warmer towards a colder pore are disregarded and thus the condensation of vapor is not computed in this study.

The sublimation rate of single pores determines the amount of heat consumed inside the CV to which this pore belongs to:

(20) $${\dot{H}}_{sub,CV}=\sum_{ij}^{}\frac{{\dot{M}}_{sub,ij}}{2}\cdot \Delta h_{sub}.$$

Equation (20) couples the mass transfer balance with the enthalpy balance. The ice phase is equally distributed in partially saturated pores. Thus, in a regular PN, the subliming pore contributes to the energy balance of both adjacent CVs, and the sublimation rate is therefore multiplied by a factor of 1/2 in Equation (20).

### 2.6. Transient Heat Transfer

The transient heat transfer problem is solved for the temperature field associated with the computational nodes and the according CVs with their distributed properties, as already discussed above. Local thermodynamic equilibrium is assumed in each CV, with *T_CV_* = *T_i_* = *const.*

The transient energy balance of each CV reads as

(21) $$V_{CV}{\left(\rho \cdot c_{p}\right)}_{CV}\frac{dT_{i}}{dt}=-\sum_{i=1}^{n}A_{CV}\cdot \lambda_{CV}\cdot \frac{T_{i}-T_{j}}{L_{CV}}-c_{p,v}\cdot {\dot{M}}_{ij}\cdot \left(T_{i}-T_{j}\right)-\Delta h_{sub}\cdot \sum_{i=1}^{n}\frac{{\dot{M}}_{sub,ij}}{2}.$$

The term on the LHS denotes the change of enthalpy inside the CV that further results in the change in its temperature. The first term on the RHS denotes heat conduction based on Fourier’s law, the second term the heat transferred by the flow of vapor (thermal convection) and the third term the heat consumed by the sublimation of ice. The numerical solution is computed by using the implicit Euler scheme [33].

The resulting set of linear equations is solved applying the above given boundary and initial conditions analog to the mass transfer problem in Equation (14). The surface pores at the top side of the PN are treated similarly as in Equation (21). The connection to the environment is given by the following condition:

(22) $${\dot{Q}}_{i\infty }={\dot{M}}_{i\infty }\cdot \left(\Delta h_{s}+c_{p}{}_{,v}\cdot T_{i}\right).$$

As the bottom CVs are in direct contact with the heating plate with constant temperature, it is realized in the same way, but anticipating solely heat conduction to the boundary pores:

(23) $${\dot{Q}}_{i,bot}=A_{CV}\cdot \lambda_{CV}\cdot \frac{T_{bot}-T_{i}}{1/2\cdot L_{CV}}.$$

### 2.7. Coupling of Heat and Mass Transfer

**Figure 5:**
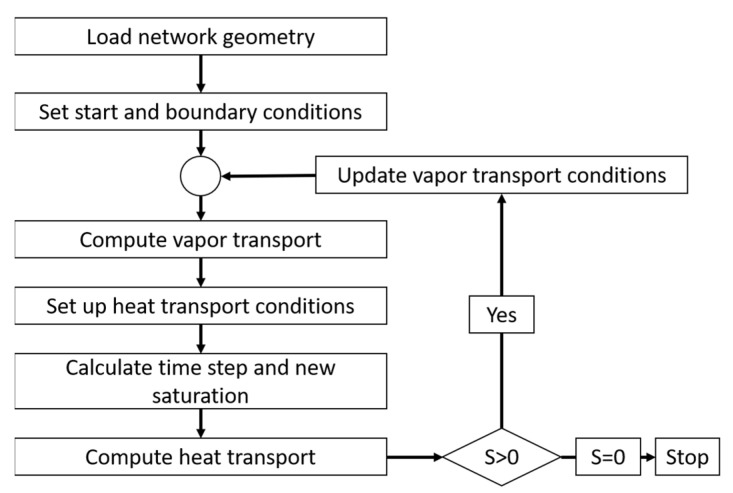
Flow chart of the primary freeze drying simulation.

The coupling of heat and mass transfer plays a critical role in the drying algorithm. The heat is consumed for the increase in temperature of the CVs (heating up) as well as for the sublimation of ice along the phase boundary (local cooling). The coupling is considered in the heat and mass balances. A flow chart of the algorithm is provided in Figure 5. As can be seen, the saturation vapor pressure, the mass transfer conditions and the thermo-physical properties are constantly updated depending on the computed temperature and pressure changes.

It is important to note that the time-constants of the kinetics of heat transfer are very different compared to the mass transfer kinetics in the regarded situation of primary freeze drying. The update of the temperature field can therefore not be coupled to the emptying of a pore, but needs to be realized more frequently. For the stability of the transient heat transfer equations it is necessary to consider the minimum time step $\Delta t=\min\left(\Delta t_{m},\Delta t_{h}\right)$, calculated from the following equations:

(24) $$\Delta t_{h}=\frac{V_{cv}\cdot {\left(\rho \cdot c_{p}\right)}_{cv}\cdot L_{cv}}{\sum \lambda_{cv}\cdot A_{cv}}$$

and

(25) $$\Delta t_{m}=\frac{V_{ice,ij}\cdot \rho_{ice,ij}}{\sum {\dot{M}}_{sub,ij}}$$

All symbols and indices used in this study are summarized in Table 4 below.

## 3. Results

### 3.1. Pore Network Saturation

**Figure 6:**
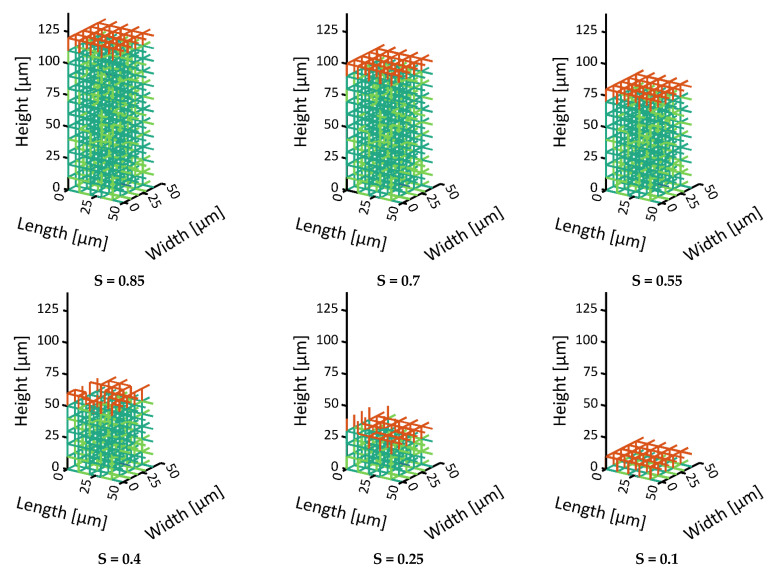
Visualization of the receding front in the monomodal PN. The colored pores follow the color code from Figure 3. The orange pores depict partially saturated pores at the sublimation front. The empty pores are not shown.

**Figure 7:**
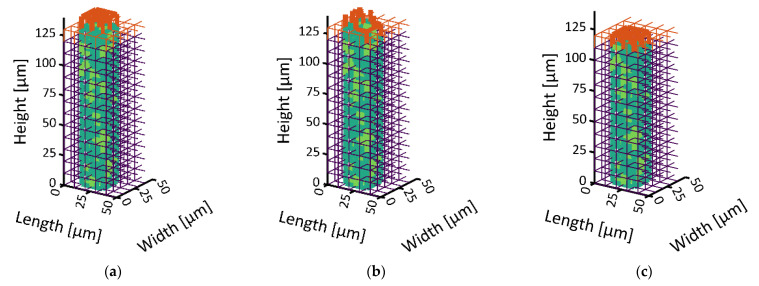
Visualization of the surface drying of the bimodal PN: (**a**) S = 0.97, (**b**) 0.94, (**c**) 0.85. The small surface pores are already dry in (**a**) and therefore not shown in this image. The large pores in the center dry slower. The front remains pinned inside the peripheral small pores (**a**–**c**) until an intermediate planar front is achieved in (**c**).

**Figure 8:**
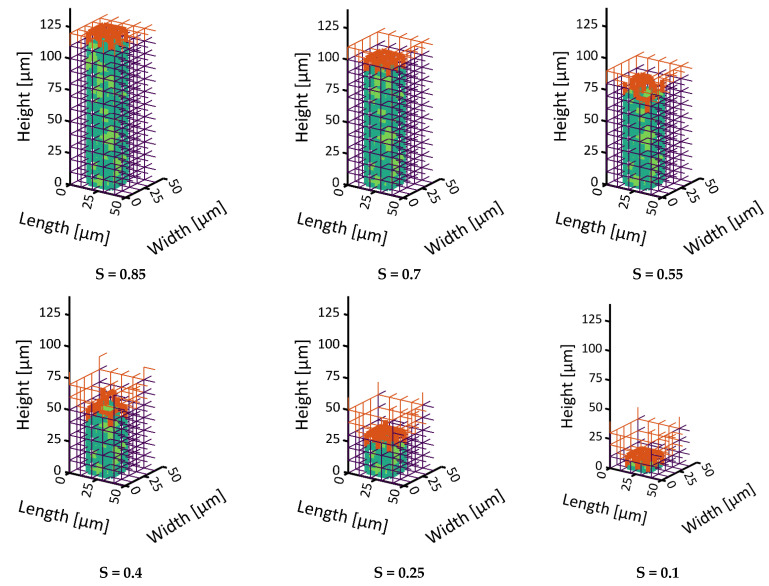
Continuation of drying from Figure 7. The larger center pores dry faster than the smaller peripheral pores resulting in an opposed front widening compared to Figure 7.

**Figure 9:**
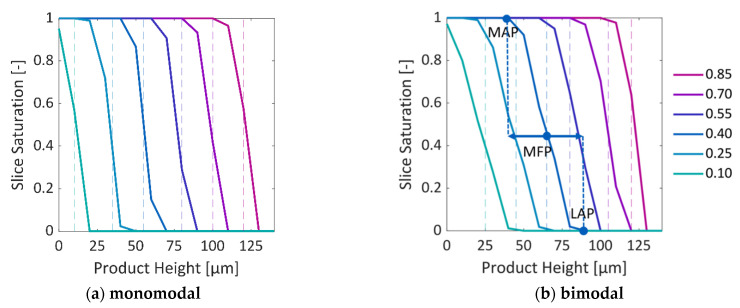
Slice saturation over the height of the PNs at the different overall saturations provided in the legend on the right. A PN slice (according to Figure 3b) includes all CV’s at a specific height, i.e., the number of slices is identical to the number of node rows of the PN. Dashed lines depict the mean front position (MFP). For the bimodal PN and S = 0.55, the most advanced point (MAP) and the least advanced point (LAP) of the front are highlighted; arrows at the MFP indicate the front width.

Freeze drying of the two investigated PNs is shown in Figure 6, Figure 7 and Figure 8. Unsurprisingly, the front of the monomodal PN is rather flat and spans only one pore layer of 10 µm width (Figure 6 and Figure 9a), which is an inherent characteristic resulting from the simplifying assumptions of the PNM. The small variation in pore sizes, as illustrated in Figure 2 above, is therefore obviously not large enough to structure the sublimation front significantly. The front width would become even smaller if the sublimation interface would be tracked within the pores. This finding supports the assumption of flat sublimation fronts usually postulated in the freeze drying literature [34].

In distinct contrast to that, the bimodal PN shows a significant widening of the sublimation front in Figure 7 and Figure 8. It reaches a maximum width of 30 µm, i.e., three pore layers when the most advanced point (MAP) of the sublimation front reaches the bottom side of the PNM (Figure 9b).

The widening occurs in two different periods. At the start of sublimation, the smaller surface pores dry out faster than the larger ones inside the surface pore row. This is explained by the significantly smaller ice content of the small pores and almost identical sublimation rates resulting from the neglection of the exact front position inside the pores. This is illustrated in Figure 7. The initial widening disappears after emptying of the top row because of the high vapor diffusion resistances inside the empty small pores that slows down the sublimation along the sides of the PNM after the first layer has dried.

After drying of the surface, the pores in the center dry faster because of the overall larger cross sections and accordingly lower mass transfer resistances. Additionally, the thermal conductivity is generally higher in the center region with larger pores. The faster drying of the large pores in the center results in the advancing of the sublimation front, whereas the slower drying of the smaller peripheral pores results in temporal front pinning. This finding is supported by experimental studies, e.g., [35], and it will be further studied in more detail in the following sections.

### 3.2. Temperature and Pressure Variation

#### 3.2.1. Spatially and Temporally Resolved Temperature Variation

The heat transfer rates at the start of drying are 5.14 mW (S = 0.996) in the monomodal PN and 2.95 mW (S = 0.965) in the bimodal PN. These values are in good agreement with the values that can be computed from the effective heat transfer coefficients, i.e., ${\dot{Q}}_{eff}=A_{\mathrm{PN}}\cdot \lambda_{\mathrm{eff}}\cdot \left(T_{\mathrm{bot}}-T_{\infty }\right)/L_{\mathrm{PN}}$ with *λ*_eff_ = 1.1731 W/m/K (monomodal) and *λ*_eff_ = 0.6844 W/m/K (bimodal) from Equations (4)–(6) and *S* = 1 (${\dot{Q}}_{eff,mono}=5.02\;\mathrm{mW}$;$\;{\dot{Q}}_{eff,bi}=2.93\;\mathrm{mW}$).

**Figure 10:**
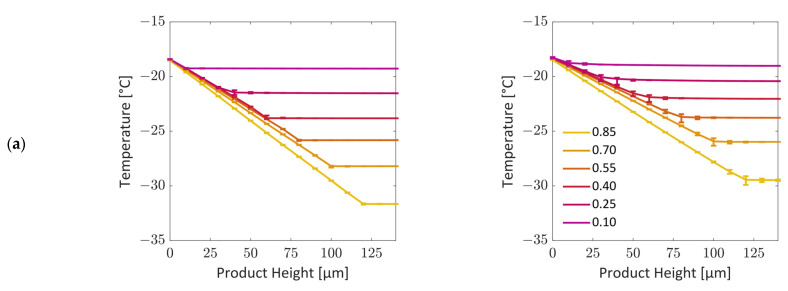

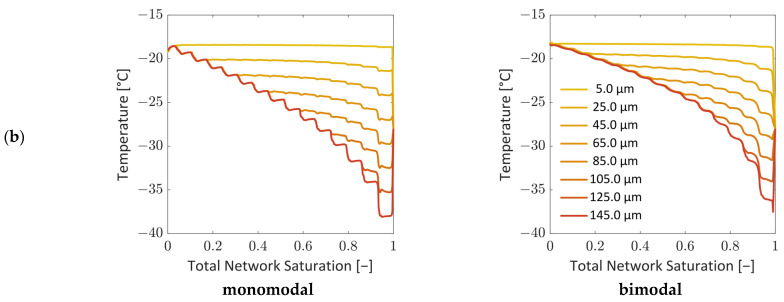
Temperature gradients: (**a**) over the height of the network at the global saturations from Figure 6 and Figure 8, bars show the minimum and maximum variation in temperature for all pores at the same height; (**b**) as a function of overall saturation for different heights.

The temperature variation of both PNs is illustrated in Figure 10 for the phase distributions shown in Figure 6 and Figure 8. The plots in Figure 10a show the spatial variation in temperature at given overall PN saturations, and the plots in Figure 10b show the variation with PN saturation at given vertical positions. In the latter case, discrete temperature jumps are clearly observed in the top slice for the case of the planar sublimation front, whereas the transition is much smoother in the case of the bimodal PN where the front widens during drying.

**Figure 11:**
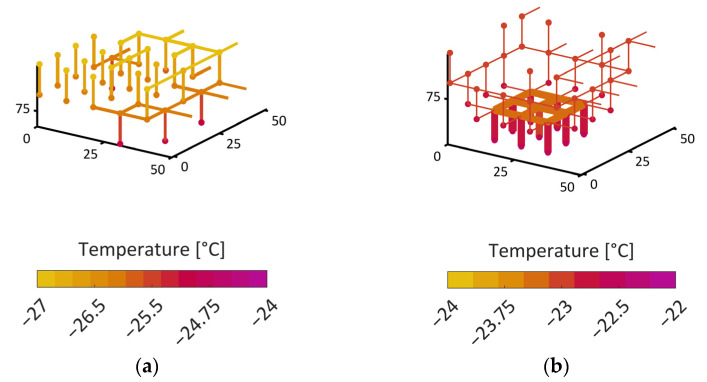
Local temperature variation at the sublimation front: (**a**) monomodal PN at S = 0.6, when the temperature variation is maximal within the front; (**b**) bimodal PN at S = 0.5, i.e., when the front has its greatest extension and the greatest temperature gradient.

The temperature value at a given product height is determined as the mean temperature of every pore at this vertical position. The temperature variation inside a slice is very low in the case of the monomodal PN. In the bimodal PN, the temperature is higher inside larger pores compared to in smaller pores. This is a result of the sublimation cooling inside the smaller pores. The temperature gradients in the horizontal direction, i.e., within a slice, are much smaller than the average temperature gradient in the vertical direction. This is illustrated in Figure 10 and Figure 11, where the local temperatures of individual pores and pore nodes are depicted for different cases of the monomodal and the bimodal PNs. The color code is associated with the temperature in this illustration.

As illustrated in Figure 10, the transition situation can be described as follows. At the beginning of freeze drying (S = 1), the temperature is set to −28 °C in both PNs (initial condition). The pores at the bottom are in contact with the heating plate and therefore initially warm up to −18 °C, whereas the pores at the top side are cooled by sublimation cooling. The achieved temperatures are −38 °C for the monomodal PN and −36 °C for the bimodal PN. Interestingly, the temperature difference between the monomodal and the bimodal PN is roughly sustained until the end of drying, where the temperatures become similar (Figure 11). This finding is surprising, since the monomodal PN has a higher effective thermal conductivity. The small difference might be explained by the slightly higher sublimation rates and the resulting higher cooling effect. Since the conditions are otherwise very similar in both cases, the comparison reflects the impact of structural variations on the freeze drying conditions.

**Figure 12:**
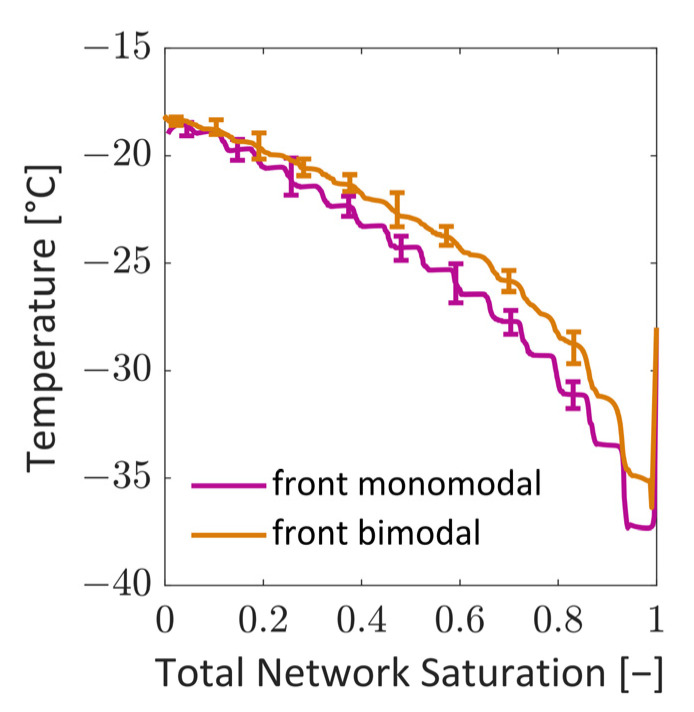
Change in the sublimation front temperatures as a function of overall PN saturation. The temperatures are computed as the mean temperature of all active pores with a sublimation interface at the given overall PN saturation. The bars represent the variation in minimum and maximum temperature along the sublimation front.

The temperature difference between the top and bottom sides results in a similar and linear temperature gradient, which is approximately 0.1 K/µm, at the start of drying in both cases. The temperature gradient decreases due to the heating up of the solid and ice phase. More clearly, the average sublimation front temperature increases during freeze drying from the aforementioned −38 °C and −36 °C to −18 °C at the end of the process. The increase in the mean slice temperature is almost linear, as presented in Figure 12. This finding is in good agreement with [4], where an increasing sublimation front temperature was also predicted for the assumption of mass-transfer-controlled freeze drying. In contrast to that, [10] predicted almost constant temperature at the sublimation interface in the case of heat-transfer-controlled primary freeze drying with purely radiative heating. Additionally, in [36], the predicted temperature at the top side was constant, whereas the bottom temperature was constantly increasing, as in our study.

At the end of drying, the PNs reach an almost isothermal state at −18 °C.

As clearly depicted in Figure 10, the linear temperature gradient is a specific property of the frozen region in this study. With the propagation of the sublimation front, the linear profile (between bottom and sublimation front, Figure 10) covers a decreasing height, because the frozen region shrinks and the dry region grows. This dry region has a constant temperature, which is equal to the temperature of the sublimation front. This is a consequence of the very small heat flow rates at the top side of both PNs. These small heat flow rates across the top surface yield almost adiabatic conditions.

#### 3.2.2. Spatially and Temporally Resolved Pressure Variations

**Figure 13:**
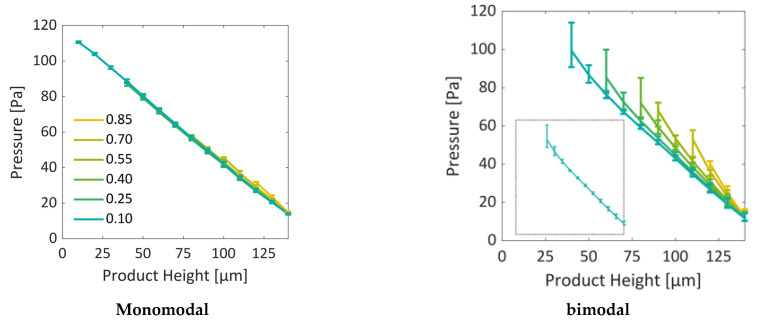
Vapor pressure gradients through the dry layers. The values are computed as slice-average values. The bars indicate the maximum and minimum values of a slice. With decreasing PN saturation, the sublimation front travels in the direction of decreasing product height, i.e., to the left in these diagrams, and is therefore found at the left end of each curve.

**Figure 14:**
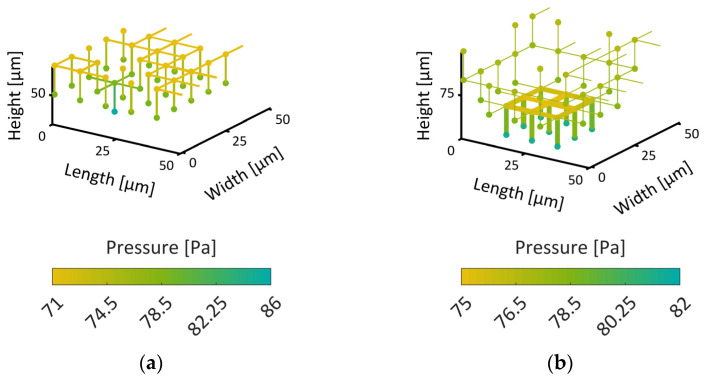
Local pressure variation at the sublimation front: (**a**) monomodal PN at S = 06; (**b**) bimodal PN at S = 0.5, i.e., when the front has its greatest extension.

Figure 13 and Figure 14 show the vapor pressure gradients inside the PNs. Similarly, as for the temperature profiles in Figure 10 and Figure 11, the given values are slice-averaged at the specified positions. As the temperature increases during drying, the saturation vapor pressure at the sublimation front increases accordingly, as shown in Figure 13. Almost linear profiles develop between the sublimation interface and the open top side, which is assigned with a constant pressure of 10 Pa in both cases. As a consequence of the overall higher temperatures in the bimodal PN, the vapor pressures are also overall higher in this situation. This results in higher vapor pressure gradients in the case of the bimodal PN. However, as will be discussed in the following section, the freeze drying rates are smaller in this case.

### 3.3. Interaction between PN Saturation, Pressure and Temperature with Thermal Properties and Mass Transfer Coefficient

#### 3.3.1. Thermal Properties

**Figure 15:**
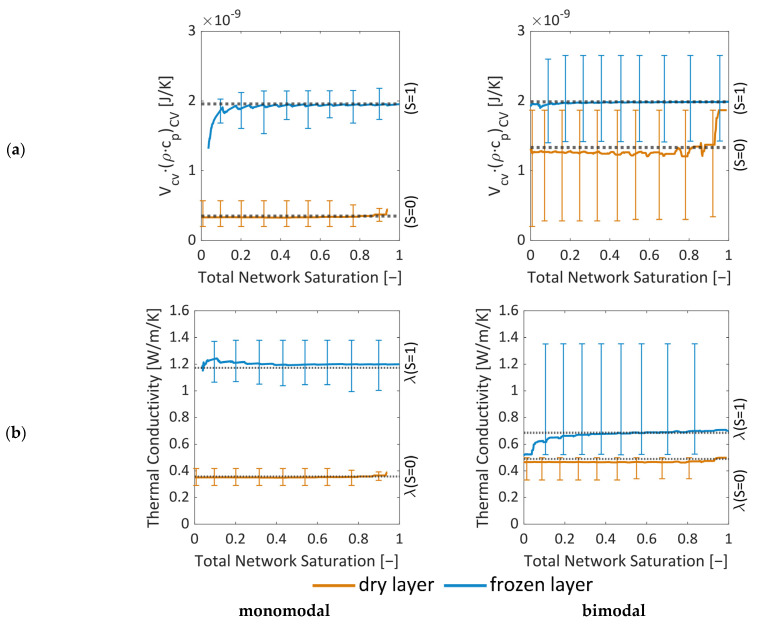
Effective properties of the CVs inside the monomodal PN (left) and the bimodal PN (right) as a function of saturation with water ice: (**a**) $\bar{V}\cdot \left(\bar{\rho }\cdot {\bar{c}}_{p}\right)$ and (**b**) thermal conductivity. The colors denote the values for the dry layer (orange) and the frozen layer (blue). The dry layer only considers pores that are completely dry (S = 0) and the frozen layer only considers pores that are full (S = 1), i.e., sublimating pores are not depicted in this figure. The error bars show the minimal and maximum values. The dashed lines indicate the average values of the completely dry or saturated PNs.

The thermal properties depend strongly on the local saturation of the PNs with water ice. This is represented by Figure 15 where the mean values of *V*_CV_∙(*ρ*∙*c*_p_)_cv_ and thermal conductivity are provided together with the maximum and minimum values (vertical lines) as functions of the overall ice saturation. The graphs illustrate the change of these parameters in the course of drying. This is especially of interest along the sublimation front, where the thermal properties can change drastically between ice-saturated and empty pores in the case of heterogeneous material structures (pore size distribution, wall thickness), as illustrated by the bars in Figure 15a,b for the bimodal PN.

Figure 15a reveals that both PNs have very similar mean values of $V_{\mathrm{CV}}{\left(\rho c_{p}\right)}_{\mathrm{CV}}$ as long as they are ice-saturated, i.e., a similar accumulation of heat can be anticipated in both cases. This changes when the CVs dry out. Then, the monomodal PNs have significantly smaller values in the dry region.

The monomodal PN has overall higher heat transfer coefficients inside the ice-saturated region (Figure 15b) and the dry layers conduct the heat slightly worse than in the case of the bimodal PN. This seems to be in contradiction to the comparison illustrated in Figure 12 but might be explained by the higher cooling effect associated with the higher sublimation rates resulting from the overall higher mass transfer rates in the monomodal PN, as will be discussed in the following.

#### 3.3.2. Mass Transfer Properties

**Figure 16:**
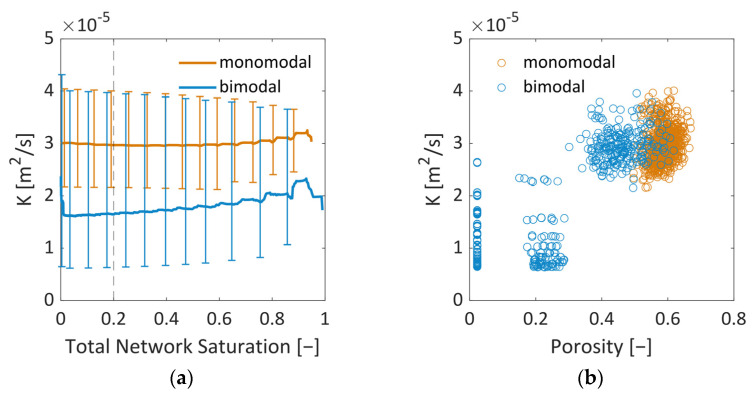
Coefficient *K* from Equation (10) in dependence of (**a**) the overall PN saturation and (**b**) the porosity of all CVs at a PN saturation of S = 0.2.

The values of *K* vary between *K* = 2.1 × 10^−5^ m^2^/s and *K* = 4.5 × 10^−5^ m^2^/s (monomodal), and *K* = 0.6 × 10^−5^ m^2^/s and *K* = 4.3 × 10^−5^ m^2^/s (bimodal) (Figure 16). This shows that, on average, the mass transfer conditions of dry pores do not vary significantly with saturation in the monomodal PN. This is different in the bimodal PN, where, at first, the small pores dry out, thereby lowering the values at the start of drying. Later on, the larger pores contribute with overall higher values. Figure 16b shows that, in the bimodal PN, three different kind of CVs exist. Control volumes that contain only large pores (in the very center of the PN) have similar mass transfer conditions as the monomodal PN. On the other hand, CVs with only small pores at the periphery of the PN show the lowest K-values; and CVs with a mixture of small and large pores have intermediate values.

For comparison, in [37], a value of *K* [m^2^/s] = 1.349 × 10^−4^ (ε/τ) d (µm) was given, assuming pressure independence of the transport coefficient and solely slip flow, which yields a value of *K* = 1.15 × 10^−4^ assuming τ = 1. This value is almost one order of magnitude larger than our values, which could be explained by the dominance of viscous flow in our model.

In summary, Figure 16 reveals higher coefficients of *K* for the monomodal PN, which allow generally higher freeze drying rates, as will be shown and discussed in what follows.

### 3.4. Drying Curve and Drying Rate Curve

#### 3.4.1. Drying Curves

**Figure 17:**
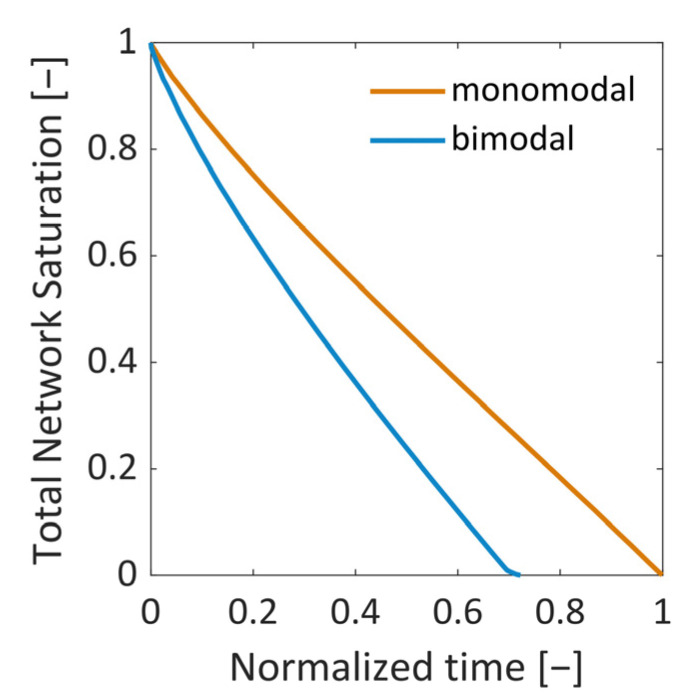
Drying curves. The bimodal PN dries 1.3 times faster than the monomodal PN because of the ice content being lower by a factor of 3.4.

The change in the overall saturation is shown in Figure 17 as a function of dimensionless time. The bimodal PN dries faster than the monomodal PN because of the significantly lower porosity and the related significantly lower overall ice content (Table 1).

#### 3.4.2. Drying Rate Curves

The drying rates of both cases are plotted together with heat- and mass-transfer-limited freeze drying from [37] in Figure 17. As already expected from the discussions related to Figure 16, the monomodal PN has a higher drying rate. It is approximately two times higher over the whole drying process. As shown in Figure 16, the coefficients of the monomodal PN can be almost twice as high as in the bimodal PN, which is, according to Equation (10) and Figure 2a, the result of the very small cross-sections of peripheral pores in the latter case.

**Figure 18:**
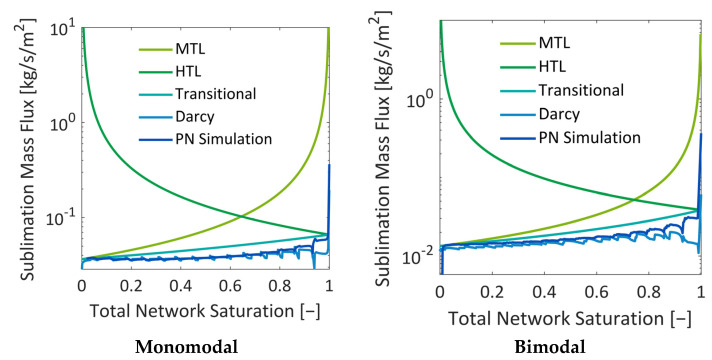
Drying rates. Comparison to freeze drying regimes in mass- and heat-transfer-limited situations from the correlations given in [37] and to the Darcy equation for vapor transport.

The referenced cases in Figure 18 are computed from [37]:

(26) $${\dot{m}}_{v,MTL}\left[kg/s/m^{2}\right]=\frac{K_{eff}\cdot {\tilde{M}}_{V}\left(P_{eq,v}\left(T_{bot}\right)-P_{\infty }\right)}{\tilde{R}T_{bot}L_{PN}}\frac{1}{1-S},$$

with *P*_eq,v_(*T*_bot_ = −18 °C) = 124.69 Pa and *P*_∞_ = 10 Pa. In Equation (26), S denotes the PN saturation; it is varied between 1 (start of drying) and 0 (end of drying); *L*_PN_ = 150 µm is the length of the PN according to Table 1. The coefficient *K*_eff_ = *K*·(ε/τ) is fitted so as to achieve an agreement with the PN simulations. It is *K*_eff_ = 5.63·10^−6^ m^2^/s = const. in the monomodal PN and *K*_eff_ = 2.06·10^−6^ m^2^/s = const. in the bimodal PN.

Furthermore,

(27) $${\dot{m}}_{v,HTL}=\frac{\lambda_{eff}\left(T_{bot}-T_{\infty }\right)}{\Delta h_{sub}L_{PN}}\frac{1}{S},$$

with *λ*_eff_ = 1.1731 W/m/K = const., estimated for the monomodal PN with the above described approach (Equations (4)–(6)) and a = 0.5, and *λ*_eff_ = 0.6844 W/m/K = const. estimated for the bimodal PN with the same approach; *T*_∞_ = −42 °C and Δ*h*_sub_ = 2838 kJ/kg according to Table 3. Figure 18 reveals that the agreement with the HTL situation is very good with the estimated heat transfer coefficients.

The transition situation is then computed from [37]:

(28) $${\dot{m}}_{v,trans}={\left[\frac{1}{{\dot{m}}_{v,MTL}}+\frac{1}{{\dot{m}}_{v,HTL}}\right]}^{-1}.$$

In addition to that, the vapor flow rates are computed from the Darcy equation:

(29) $${\dot{m}}_{v,Da}\left[kg/sm^{2}\right]=\frac{K_{eff}\cdot {\tilde{M}}_{V}\left(P_{eq,v}\left(T_{front}\right)-P\right)}{\tilde{R}T_{front}\left(L_{PN}-z_{front}\right)}.$$

In this equation, the following parameters are used: *T*_front_ according to Figure 12 and *P*_eq,v_ computed from Equation (18) with *T*_front_; *P* = 10 Pa, *L*_PN_ = 150 µm and *z*_front_ = [150:0] µm. In this computation, *K*_eff_ = 4.7 ∙ 10^−12^ m^2^/s. As shown in Figure 18, the agreement is very good except for at the start of drying, where the Darcy equation yields generally lower values due to the transient temperature changes at the start of the simulation. The high temperature gradients inside the PN cannot be realized by the Darcy equation. The otherwise good agreement between the curve from Equation [30] and the Darcy equation reflects the fact that the mass flow regime can be expected in the Darcy regime rather than in Knudsen or transition regimes in the studied cases.

Figure 18 shows that the drying rates in both PNs drop until approximately S ≈0.99 in both situations. This is explained with the initial PN temperature, which is much higher than in the environment, namely *T* = −28 °C, and the relatively high vapor conductivity of the surrounding bulk of *g*_∞_ = 1∙10^−12^ m∙s = const. This yields a relatively high pressure difference of Δ*P*_v_ = *P*_v_(−28 °C) − *P*_v_(−42 °C) = 36.5 Pa at the start of drying. This, together with the sublimation front being located at the very top side of the PNs, results in very high initial drying rates. The drying rates drop when the surface pores begin to dry out and when the sublimation front recedes from the top side accordingly. The initial conditions could be adjusted in the future to achieve a better agreement with the transition case by reducing the vapor pressure difference between the relatively warm PN surface (at −28 °C) and the surroundings (at −42 °C), as well as by adjusting the vapor transport coefficient *g*_∞_.

Apart from the initial differences, the agreement with Equation (28) is very good. The deviation is lower in the case of the monomodal PN because of the lower spread of transport coefficients, whereas the drying rate varies more in the case of the bimodal PN, resulting in higher deviations from cases in the literature. A similar observation can be found for the comparison with the Darcy equation. Apart from the initial deviation, the agreement is very good for the monomodal PN because *K_eff_* is almost constant in this case (Figure 16). However, in the bimodal PN, the mixed population of large and small pores results in a significant variation in *K_eff_* wherefore the situation cannot be captured with the same satisfaction by the Darcy equation in the bimodal case. Secondly, the position of the sublimation front is determined as an average value in this case, which can result in further deviations.

In summary, Figure 18 illustrates that the overall agreement with the transition situation in [37] and the Darcy equation is very good in the case of a monomodal PN. However, in the case of heterogeneous pore space, with distributed transport coefficients, the curves cannot be fitted to the PN simulation results with the same agreement. This reflects the impact of the structural material properties on the computable freeze drying regimes as well as the relevance of the pore-scale modeling approach for such situations. An even stronger impact is expected if the PN simulation were conducted with reconstructed image data [21].

## 4. Conclusions

In this paper, a non-isothermal pore network (PN) model of primary freeze drying with quasi-steady vapor transport and transient heat transfer was presented. To the best of our knowledge, this is the first PN model of freeze drying. It was realized with constant temperature boundary conditions at the top and bottom sides and periodic boundary conditions along the vertical interfaces. The advantage of the PN model is the representation of the continuous void space by discrete pores, which allows one to set-up and solve the mass and enthalpy balances in a discrete way with relatively low computational effort. This makes it possible to study the impact of the porous structure on transport conditions, as well as the evolution and progress of the sublimation front.

Simulation results were therefore presented for two significantly different situations. In the first case, a homogeneous PN with monomodal PSD was considered, and in the second case, a PN with local variation in pore structure was studied. In the latter case, pores along the vertical sides of the PN were smaller by a factor of 5 than the pores in the center. The first scenario revealed a roughly planar sublimation front, in good agreement with literature references, e.g., [34]. The second situation instead showed the formation of distinct structures of the sublimation front, similar to what has already been observed in experiments. At first, the smaller pores dried faster and yielded a sublimation front which was slightly curved downwards at the sides, i.e., it had a convex shape. This was explained by the high initial mass transfer rates, which were achieved by the high vapor pressure gradients between the PN and the surroundings, as well as the high vapor transport coefficients at the top side of the PN, and the low ice content in the small pores. Once the first row of pores was dried and higher mass transfer resistances occurred inside these pores, the progress of the sublimation front slowed down in this region of the PN. In contrast, the faster drying of the larger pores in the center finally resulted in a concave shape of the sublimation front. The distance between the LAP and the MAP continuously increased until the first pore in the bottom layer dried out. The maximum width of the sublimation front was limited by the small PN height in this study. It is expected that it depends on the spatial variation in the PSD, which could prospectively be studied in larger PNs, where the extension of the sublimation front width would not be limited by the small height of the PN.

In summary, the influence of the pore structure on the shape of the sublimation front could be nicely demonstrated in this study, although the mass transfer was found to be in the Darcy regime. It could be shown that the increase in mass transfer resistances due to the presence of smaller pores can reduce the overall drying rates in the case of the bimodal PN. This was a major effect in this study. The variation in the thermal properties did indeed change the PN temperatures slightly in the studied cases, but this seemed to not impact strongly on the drying behavior.

The comparison of the sublimation rates with references from the literature showed an overall good agreement, especially for the monomodal PN. However, it also revealed that the pore-scale structure variations and the associated spread of transport parameters (heat and mass) results in stronger deviations. These deviations are expected to become even stronger when the structure is more distributed, e.g., in PNs reconstructed from real porous structures [21]. Consequently, after the successful demonstration of the new mathematical approach on regular PN lattices in this paper, the algorithm shall be adopted for the simulation of reconstructed porous materials [21] in the next step.

## Figures and Tables

**Table 1:** Geometric properties of the PNs used in this study.

Parameter	Monomodal	Bimodal
Pore number [−]	1100	1100
Computational nodes [−]	375	375
Size of the domain [µm]	50 × 50 × 150	50 × 50 × 150
Total initial ice volume [µm^3^]	21.70 × 10^4^	6.53 × 10^4^
Porosity [−]	0.58	0.17
Pore diameter [µm]	5 ± 0.2	5 ± 0.2/1 ± 0.2
Pore length [µm]	10	10
Length of CVs [µm]	10	10
Cross sectional area of PN [µm^2^]	2.5 × 10^3^	2.5 × 10^3^
Volume of a CV [µm^3^]	1 × 10^3^	1 × 10^3^

**Table 2:** Overview of process parameters. The chamber temperature was calculated from Equation (18) for $P_{\infty }$.

	Parameter	Symbol	Value	Unit
Boundary conditions	Chamber pressure	$P_{\infty }$	10	Pa
	Chamber temperature	$T_{\infty }\left(P_{\infty }\right)$	−42	°C
	Bottom temperature	$T_{Bot}$	−18	°C
Initial conditions	Temperature in PN	$T_{PN}$	−28	°C
	Pressure in PN	$P_{PN}$	10	Pa
	Ice saturation	*S*	1	-

**Table 3:** Overview of material parameters as mean values for the given temperature ranges. Heat capacity of maltodextrin was measured with differential scanning calorimetry (Setaram DSC 92). Density of maltodextrin was measured with helium pycnometry (Micromeritics AccuPyc 1330).

Parameter	Symbol	Value	Unit
Ice density	$\rho_{ice}$(−15 to −30 °C)	919 [27]	kg/m^3^
Solid density	$\rho_{s}$(20 °C)	1565	kg/m^3^
Thermal conductivity of ice	$\lambda_{ice}$(−15 to −30 °C)	2.42 [28]	W/m/K
Thermal conductivity of solid	$\lambda_{s}$(0 °C)	0.5 [29]	W/m/K
Heat capacity of ice	$c_{p,ice}$(−10 to−40 °C)	1930 [30]	J/kg/K
Heat capacity of solid	$c_{p,s}$(0 to −30 °C)	1250	J/kg/K
Heat capacity of vapor	$c_{p,v}$(−10 to −40 °C)	1617 [30]	J/kg/K
Molecular weight of vapor	${\tilde{M}}_{v}$	18.02	kg/kmol
Viscosity of vapor	*η* (T)	18.4558·10−7(T1.5(T+650)) [30]	Pa∙s
Sublimation enthalpy of ice	$\Delta h_{sub}$(−15 to−35 °C)	2838 [31]	kJ/kg

**Table 4:** Symbols and indices used in this study.

Symbol	Name	Unit	Index	Name
a	Krischer fitting factor	-	Bot	Bottom
A	Area	m^2^	bi	Bimodal
b	Vector of boundary conditions	-	CV	Control volume
c_p_	Heat Capacity	J/K	Da	Darcy
d	Diameter	m	eff	Effective
ε	Porosity	-	eq	Equilibrium
g	Vapor conductivity	m∙s	front	Front
$\dot{H}$	Enthalpy flow	J/s	h	Heat
Δh	Enthalpie	J/kg	HTL	Heat transfer limited
K	Mass transfer coefficient	m/s^2^	i,j,m,n,k	Computational nodes
Kn	Knudsen Number	-	∞	Infinity
L	Length	m	ice	Ice
λ	Thermal conductivity	W/m∙K	m	Mass
$\tilde{M}$	Molecular weight	mol/kg	mono	Monomodal
$\dot{M}$	Mass flow	kg/s	MTL	Mass transfer limited
η	Viscosity	Pa∙s	PN	Pore Network
P	Pressure	Pa	par	Parallel
$\dot{Q}$	Heat flow	J/s	s	Solid
r	Radius	m	sub	Sublimation
$\tilde{R}$	Gas constant	J/kg∙K	ser	Series
ρ	Density	kg/m^3^	*	Saturated
S	Saturation	-	trans	Transition
t	Time	s	v	Vapor
T	Temperature	°C/K	void	Void
V	Volume	m^3^		
z	Length	m		

## Data Availability

Not applicable.
